# Quality of residential facilities in Italy: satisfaction and quality of life of residents with schizophrenia spectrum disorders

**DOI:** 10.1186/s12888-022-04344-w

**Published:** 2022-11-18

**Authors:** Alessandra Martinelli, Helen Killaspy, Cristina Zarbo, Sara Agosta, Letizia Casiraghi, Manuel Zamparini, Fabrizio Starace, Matteo Rocchetti, Giovanni de Girolamo, Mirella Ruggeri, DIAPASON consortium, Stefano Barlati, Maria Elena Boero, Giancarlo Cerveri, Massimo Clerici, Giulio D’Anna, Antonio De Novellis, Vittorio Di Michele, Pasquale Di Prisco, Federico Durbano, Federico Facchini, Lucio Ghio, Patricia Giosuè, Carmelo Greco, Valeria Latorre, Emanuela Leuci, Daniela Malagamba, Antonio Maone, Marina Marina, Annalisa Maurizi, Emiliano Monzani, Roberto Placenti, Luca Rancati, Arturo Rippa, Chiara Rovera, Andrea Silva, Giambattista Tura, Stefano Zanolini

**Affiliations:** 1 Section of Psychiatry, Verona Hospital Trust, AOUI, Verona, Italy; 2 Unit of Clinical Psychiatry, IRCCS Istituto Centro San Giovanni di Dio Fatebenefratelli, Via Pilastroni, 4, 25125 Brescia, BS Italy; 3 Division of Psychiatry, University College London, London, UK; 4 Unit of Epidemiological and Evaluation Psychiatry, IRCCS Istituto Centro San Giovanni di Dio Fatebenefratelli, Brescia, Italy; 5 Mental Health and Dependence, AUSL of Modena, Modena, Italy; 6 Clinical Psychology Unit, ASST of Mantua, Mantua, Italy; 7 Department of Mental Health and Dependence, ASST of Pavia, Pavia, Italy

**Keywords:** Schizophrenia, Residential facilities, Recovery, Quality of care, Quality of life, Functioning

## Abstract

**Background:**

Recovery and human rights promotion for people with Schizophrenia Spectrum Disorders (SSDs) is fundamental to provide good care in Residential Facilities (RFs). However, there is a concern about rehabilitation ethos in RFs. This study aimed to investigate the care quality of Italian RFs, the quality of life (QoL) and care experience of residents with SSD.

**Methods:**

Fourty-eight RFs were assessed using a quality assessment tool (QuIRC-SA) and 161 residents with SSD were enrolled. Seventeen RFs provided high intensity rehabilitation (SRP1), 15 medium intensity (SRP2), and 16 medium-low level support (SRP3). Staff-rated tools measured psychiatric symptoms and psychosocial functioning; user-rated tools assessed QoL and satisfaction with services. RFs comparisons were made using ANOVA and Chi-squared.

**Results:**

Over two-thirds patients (41.5 y.o., SD 9.7) were male. Seventy-six were recruited from SRP1 services, 48 from SRP2, and 27 from SRP3. The lowest QuIRC-SA scoring was Recovery Based Practice (45.8%), and the highest was promotion of Human Rights (58.4%). SRP2 had the lowest QuIRC-SA ratings and SRP3 the highest. Residents had similar psychopathology (*p *= 0.140) and functioning (*p *= 0.537). SRP3 residents were more employed (18.9%) than SRP1 (7.9%) or SRP2 (2.2%) ones, and had less severe negative symptoms (*p *= 0.016) and better QoL (*p *= 0.020) than SRP2 residents. There were no differences in the RF therapeutic milieu and their satisfaction with care.

**Conclusions:**

Residents of the lowest supported RFs in Italy had less severe negative symptoms, better QoL and more employment than others. The lowest ratings for Recovery Based Practice across all RFs suggest more work is needed to improve recovery.

**Supplementary Information:**

The online version contains supplementary material available at 10.1186/s12888-022-04344-w.

## Introduction

In the last three decades, in Europe, there has been a gradual decrease in the number of mental hospital beds and a parallel development of community-based care, which has enabled many people with Severe Mental Disorders (SMD) to live independently in the community or accommodation with a variable degree of support [1, 2]. Since 2005, the European Commission’s Green Paper [3] and the United Nations Convention on the Rights of Persons with Disabilities [4] have recommended the respect of human rights and fundamental freedom of people with any kind of disability. According to the Mental Health Declaration of Europe [5] and the NICE [6], the care of people with a psychiatric disability should be implemented mostly in community settings, should be recovery-oriented, flexible and involve all stakeholders. The provision of recovery-based practice and the promotion of people’s human rights have been found to be positively associated with successful rehabilitation for people with severe and complex mental health problems in England [7, 8]. Every person, despite their psychiatric and social impairments, deserves the opportunity to live the most satisfying life as possible, being integrated into the community, and should have the possibility to carry out significant activities [9, 10], covering all adulthood roles at home, work, school or in other social areas [11].

In Italy, since 1978, all mental hospitals have been closed [12, 13] and replaced with a range of community-based services (community mental health centers, general hospital psychiatric wards and day-care centers [14, 15]). For those patients with more severe functional impairment, residential care delivered by community Residential Facilities (RFs) or other forms of supported accommodation has been provided [16–18]. Commonly, RFs represent a fundamental component of rehabilitation programmes for people with SMD. RFs aim to support them to learn or re-learn daily living skills and gain confidence to achieve personal recovery, be socially included, and live as independently as possible despite their disability [19–21].

The most recent data provided by the Italian Ministry of Health show that RFs account for about 40% of the total Department of Mental Health costs, despite involving only 3.4% of all patients in treatment [22] (28,895 patients hosted in 2220 RFs). Half of the residents of Italian RFs are patients with a diagnosis of Schizophrenia Spectrum Disorders (SSDs) [23]. Although RFs are regulated by Italian national guidelines [24, 25], they are somewhat heterogeneous in their approach with differing aims, rules, size, staffing, length of stay, environmental features, and target population [26–28]. According to the Italian Ministry of Health, RFs can be broadly categorized into five main types and two dimensions: rehabilitation intensity and care intensity [24] (see Table 1). Italian RFs aim to support residents to progress from more supported settings to more independent settings as they gain competences [24, 29]. The care pathway is thus organised so that people move on from RFs providing higher support (Table 1 types SRP1 and SRP2) to those providing intermediate support (SRP3.1) and then to those with minimal support (SRP3.3). Of note, SRP3.2 is designed for residents with severe but stable mental health problems who are less likely to be able to progress to a more independent setting [2, 30, 31].

**Table 1:** Classification of Italian mental health residential facilities (RFs) according to Ministry of Health typology

CLASSIFICATION	DESCRIPTION
*SRP1* *High intensity rehabilitation*	High-intensity support and high-intensity rehabilitation Target: people with complex mental health needs with severe and unstable psychopathology and low global functioning Aim: to stabilize or maintain stable psychopathology and functioning Staff on-site 24/7 Max length of stay 18 months
*SRP2* *Medium intensity rehabilitation*	High-intensity support and medium-intensity rehabilitation Target: people with complex mental health needs with severe and unstable psychopathology and low-moderate global functioning Aim: to stabilize or maintain stable psychopathology and functioning Staff on-site 24/7 Max length of stay 36 months
*SRP3.2* *High level support*	High-intensity support and low-intensity rehabilitation Target: people with long-term stable and chronic mental health psychopathology, low global functioning, and high care needs Aim: to maintain psychopathology and functioning Staff on-site 24/7 Max length of stay regionally established
*SRP3.1* *Medium level support*	Medium-intensity support and medium-intensity rehabilitation Target: people with severe but stable psychopathology with moderate-low personal and social functioning Aim: to support the person to gain community living skills Staff on-site 12 h a day Max length of stay regionally established
*SRP3.3* *Low level support*	Low-intensity support and medium-intensity rehabilitation Target: people with stable psychopathology and moderate-good functioning Aim: to support independence and self-management with monitoring Visiting support few hours a day or a week Max length of stay regionally established

*SRP* Struttura Residenziale Psichiatrica/ Psychiatric Residential Facility

Despite the important role of RFs in community care provision, few studies have investigated their quality and their association with residents’ outcomes [31–38]. Therefore, the objectives of this study were to:

- Investigate the quality and characteristics of RFs in Italy using the *Quality Indicator for Rehabilitative Care - Supported Accommodation (QuIRC-SA)* [39];
- Investigate sociodemographic and clinical characteristics (i.e., severity of the disorder, psychosocial functioning) and experiences of care (i.e., perceived RF’s atmosphere and satisfaction with the service) of residents of RFs with a diagnosis of SSD.

## Methods

### Procedure and participants

This cross-sectional study is part of the national project “*DAily time use, Physical Activity, quality of care and interpersonal relationships in patients with Schizophrenia spectrum disorders (DiAPASon)”* [40]. Enrolment of RFs occurred from September 2020 to April 2021.

**Fig. 1:**
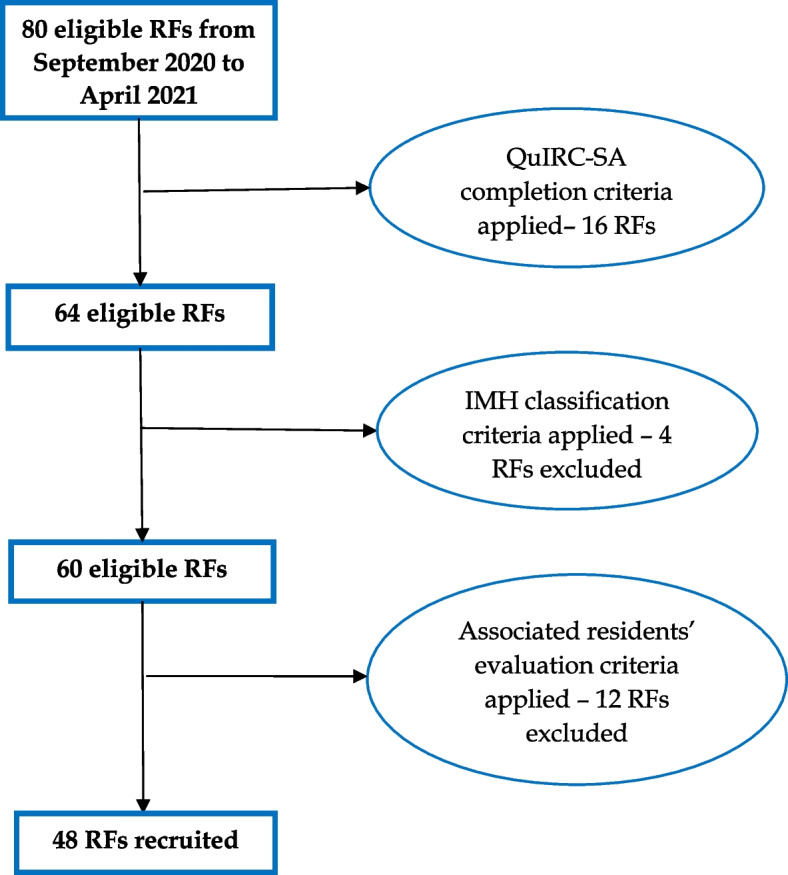
Flow-chart showing process of recruitment of the RFs

A total of 80 RFs agreed to participate in the DiAPAson study. Sixteen RFs were excluded because they did not complete the QuIRC-SA, four because they were not RFs categorizable according to the Italian Ministry of Health classification (one was a nursing home, one a facility for people with cognitive and/or physical disability, two were forensic facilities) and 12 because they did not enrol at least one resident with SSD. Therefore, 48 RFs (60.0%) were included in this study (see Fig. 1).

Participating RFs were located in different parts of Italy: 39 (81.3%) in Northern Italy, 3 (6.3%) in the Centre and 6 (12.5%) in Southern Italy. RFs were categorized according to the Italian Ministry of Health classification (see Table 1). Of the five types of RF only those which are not formally classified as ‘rehabilitation’ (SRP3.2) were not recruited. The categories SRP3.1 and SRP3.3 were merged (‘SRP3’) for this study given their similarities (Table 1). The 48 RFs participating in the project were equally distributed between the following three categories: 17 (35.4%) SRP1, 15 (31.3%) SRP2 and 16 (33.3%) SRP3.

The Principal Investigators of the *DiAPAson project* agreed on participant eligibility criteria to address the specific aims of the broader research programme. These are fully detailed in the study protocol [40]. The eligibility criteria for residents were:

- having a diagnosis of SSD according to DSM-5 [41],
- Age 20-55 years;
- MMSE score equal to or higher than 24;
- adequate understanding of the Italian language to participate in a research interview;
- willingness to complete a range of clinical measures.

Exclusion criteria were:

- lacking the capacity to provide informed consent (e.g. because of low education or cognitive impairment);
- a current DSM-5 co-morbid diagnosis of a substance use disorder, or history of clinically significant head injury or cerebrovascular/neurological disease [41].

Within participating RFs, there were a total of 676 places with occupancy of 79.6%: approximately 538 residents were potentially available for the study. Of these, a proportion did not meet eligibility criteria. Overall, 161 residents living in the 48 recruited RFs met the criteria and were involved in this part of the DiAPAson study: 76 (47.2%) residents of SRP1 services, 48 (29.8%) residents of SRP2 services and 37 (23.0%) residents of SRP3 services. Standardised tools were completed as described below, with all measures for all participants being completed within 1 month in each RF.

### Measure and variables

#### RF quality

The RF senior manager completed the Italian version of the QuIRC-SA (https://quirc.eu/quirc-sa/) [39]. The QuIRC-SA consists of 143 items, of which 55 provide descriptive data on service characteristics and 88 contribute to scores on seven domains that assess different aspects of the quality of care provided: living environment (this is not completed for non-building based facilities with very low support i.e., where the resident lives in an independent apartment and has visiting support), therapeutic environment, treatments and interventions, self-management and autonomy, social interface, human rights, and recovery-based practice. The quality of each domain is evaluated as a percentage, with higher scores denoting better quality.

#### Resident evaluations

- Socio-demographic and clinical data were obtained by the researchers using face to face interviews with RF staff and residents and corroborated, when required, through case note review. The staff completed the following standardised measures *Severity of psychopathology* was assessed with: (a) the Brief Psychiatric Rating Scale (BPRS), which consists of 24 items each rated on a seven-point Likert scale (from 1 = no symptom to 7 = extremely severe symptom) [42–44]; (b) the Brief Negative Symptom Scale (BNSS) which comprises 13 items, each rated on a six-point Likert-scale (0 = no symptom, 6 = severe symptom) [45, 46];
- *Psychosocial functioning* was assessed with the Specific Levels of Functioning Scale (SLOF) that indicates the level of support the participant needs to perform each task and consists of 43 items. Each of the questions is rated on a 5-point Likert scale, with an overall total score ranging from 43 to 215. A higher total score indicates a higher psychosocial functioning [47, 48].

The residents completed the following self-rated validated instruments:

- *Functioning/disability* was assessed using the WHO Disability Assessment Schedule (WHODAS 2.0) which comprises 12 items, each rated on a five-point Likert scale (0 = no difficulty; 4 = extreme difficulty). A higher score reflects a higher disability [49].
- *Quality of life was* assessed with the WHO Quality of Life-Brief scale (WHOQOL-Brief) which comprises 26 items that cover four domains (physical health, psychological health, social relationships, and environmental conditions), with each item rated on a five-point Likert scale (1 = not at all satisfied; 5 = extremely satisfied). A total score is calculated by summing the scores of all 26 items, with an overall range from 0 to 100. Higher scores reflect better quality of life [50];
- *Therapeutic milieu of the RF* was evaluated using the Ward Atmosphere Scale-patient version (WAS-P), which comprises 100 short statements describing various aspects of the facility, each of which can be answered as True (1) or False (0), providing a total score between 0 to 90. The items are then grouped into 10 subscales that in turn refer to three general dimensions: Relationships, which assesses the involvement of the individuals in the running of the service and with one another; Treatment Program, which assesses the nature of the care delivered; and System Maintenance, which assesses how well the service helps the individual’s personal growth and recovery. Higher scores reflect better quality of the therapeutic environment [51, 52];
- *Satisfaction with care* was assessed using the Verona Service Satisfaction Scale (VSSS-32) which comprises 32 items, each rated on a five-point Likert scale (1 = not at all satisfied; 5 = extremely satisfied). The items are grouped into 7 subscales (overall satisfaction, professionals’ skills, and behaviour, efficacy, types of intervention, information, access, relatives’ involvement) [53].

### Statistical analysis

Simple descriptive statistics were generated for the three types of RF (SRP1, SRP2, SRP3). For continuous variables we used means, medians, standard deviations, and ranges. The Kolmogorov-Smirnov and Shapiro-Wilk tests were used to examine whether data were normally distributed. Comparisons were made between the three types of RF using ANOVA with post hoc Bonferroni corrections for continuous variables. For non-normally-distributed variables, non-parametric Kruskal-Wallis tests were used. Categorical variables were compared using Chi-Square tests. All statistical tests were two-sided and a *p*-value ≤0.05 was considered significant. All statistical analyses were performed using the SPSS version 27.0.

## Results

### RF quality of care

Table 2 shows the results of the quality assessment of RFs evaluated with the QuIRC-SA. The total mean QuIRC-SA score across all RFs was 52.3% (SD 9.3). The mean scores across all RFs were lower than 50% for the Social Interface domain (48.6%, SD 11.4) and Recovery Based Practice domain (45.8%, SD 9.1). The QuIRC-SA domain with the highest mean score across RFs was the promotion of Human Rights (58.4, SD 11.6).

**Table 2:** Quality of the performance of RFs in each QuIRC-SA domain by RF type

QuIRC-SA domain (range 0 - 100%) Higher scores denote better quality	SRP1 ***N =*** 17 (35.4%) RFs	SRP2 ***N =*** 15 (31.3%) RFs	SRP3 ***N =*** 16 (33.3%) RFs	Total RFs ***N =*** 48 (100%)	p ANOVA*	Bonferroni post-hoc correction
Living environment, Mean (SD)	58.3 (9.3)	58.4 (11.2) (14 RFs)	–	58.3 (10.0)	0.979	–
Therapeutic environment, Mean (SD)	52.9 (8.9)	49.8 (6.0)	50.7 (5.8)	51.2 (7.1)	0.447	–
Self-management and autonomy, Mean (SD)	49.5 (6.2)	45.4 (6.9)	58.1 (9.3)	51.1 (9.1)	**< 0.001**	SRP1/SRP2 vs SRP3
Social interface, Mean (SD)	50.9 (9.1)	41.4 (11.1)	52.9 (11.2)	48.6 (11.4)	**0.008**	SRP2 vs SRP1/SRP3
Human rights, Mean (SD)	53.2 (8.5)	56.9 (13.2)	65.2 (10.1)	58.4 (11.6)	**0.008**	SRP1 vs SRP3
Treatments and interventions, Mean (SD)	57.7 (6.0)	53.8 (5.1)	56.8 (5.5)	56.2 (5.7)	0.143	–
Recovery based practice, Mean (SD)	46.2 (10.1)	42.1 (7.6)	48.8 (8.6)	45.8 (9.1)	0.115	–
Total mean (SD) score	52.7 (10.9)	47.9 (5.4)	57.3 (7.0)	52.3 (9.3)	**< 0.001**	SRP2 vs SRP1 vs SRP3

*SRP1* High intensity rehabilitation, *SRP2* Medium intensity rehabilitation, *SRP3* Medium-low level support

*SRP* Struttura Residenziale Psichiatrica/ Psychiatric Residential Facility

*Bold values indicate statistical significance at the *p* < 0.05 level

Overall, SRP2 services had lower QuIRC-SA domain scores than the other types of RF, except for the Human Rights domain, whereas SRP1 services had higher scores for the Therapeutic Environment (52.9%, SD 8.9), Treatments and Interventions (57.7%, SD 6.0) and Living Environment (58.3%, SD9.3) domains. However**,** quality of care was significantly higher for SRP3 services compared to other types of RF in the following domains: Self-Management and Autonomy (F = 31.2, df 3, *p* < 0.001), Social Interface (F = 13.8, df 3, *p* = 0.008), and Human Rights (F = 15.6, df 3, *p* = 0.008).

### Characteristics of residential facilities

As shown in Supplementary Table 1, the current model of care had been used in the RFs for an average 15.5 (SD = 8.5) years. The majority of SRP1 services were located in the suburbs, while most SRP2 and SRP3 services were in the inner city. All the RFs included in this study hosted both male and female residents. Most bedrooms were single (70.8%) and there were relatively high levels of occupancy (percentage of currently filled places/total places: 79.8%).

#### Staffing

Most RFs had an allocated psychiatrist (89.6%), and each resident also had a named psychiatrist with the local community-based mental health service (sometimes this was the same psychiatrist). Most RFs employed support workers (81.3%), nurses (79.2%) and clinical psychologists (72.9%) amongst their staff. More than half of the RFs (56.3%) had a psychiatric health worker (‘TeRP’) (specifically trained in mental health rehabilitation with the main focus on the development of vocational skills), half had a social worker (50.0%), and nearly one third (29.2%) reported that they provided some sort of psychotherapy to residents. Only 9 RFs (19.1%) had access to a specialist vocational therapist (e.g., an employment support worker such as someone trained in the Individual Placement and Support approach who supports residents to seek and sustain open employment). Five RFs (10.4%) employed former RF residents as staff members (Supplementary Table 1).

#### Rehabilitation programme

Almost all RFs (97.9%) used individualised care plans and provided a named keyworker for each resident (who acted as the main contact for family members and other carers and drew up care plans with the resident). Families were actively involved with more than half of the residents’ care (53.9%) (Supplementary Table 1).

As shown in Supplementary Table 1, RF managers reported an average expected maximum length of stay of 2.7 years (SD 1.0), ranging from 1 to 5 years, while the actual average length of stay was 2.9 years (SD 1.0), ranging from 2 to 6 years. Managers reported that a mean 4.9 (SD 5.0) of their residents had moved to more independent accommodation in the last 2 years, and this was more likely for those living in SRP1 services (7.2, SD 7.0) than SRP3 (2.5, SD 2.1) services (F = 0.0, df 2, *p* = 0.025). Managers reported that they hoped that around a third (31.3%) of their service users would move to more independent accommodation over the next 2 years, although they expected that fewer (21.9%) would do so.

### Sociodemographic and clinical characteristics, quality of life and subjective rating of the therapeutic environment of RF users

As shown in Table 3, most patients were male (70.4%), with a mean age of 41.5 years (SD 9.7) and had spent a mean of 39.1 months (SD 53.8) in the RF; the duration of stay was significantly longer for those in SRP3 than SRP1 services (F = 6.307, df 2, *p* < 0.001). Most residents (81.1%) had no regular employment.

**Table 3:** Sociodemographic and clinical characteristics of residents of RFs by RF type

	SRP1 ***N =*** 76 (47.2%) residents	SRP2 ***N =*** 48 (29.8%) residents	SRP3 ***N =*** 37 (23.0%) residents	Total ***N =*** 161 (100%) residents	p ANOVA or Chi-Square* tests	Bonferroni post-hoc correction
**Sex**						
Male*	52 (68.4%)	38 (82.6%)	22 (59.5%)	112 (70.4%)	0.062	–
**Mean (SD) age (years)**	39.8 (9.9)	43.1 (9.5)	43.1 (9.4)	41.5 (9.7)	0.101	–
Marital status*						
*Single (including widowed/separated)*	75 (98.7%)	44 (95.7%)	33 (89.2%)	152 (95.6%)	0.070	–
**Mean (SD) length of education (years)**	11.9 (3.8)	11.6 (3.4)	11.8 (2.9)	11.8 (3.5)	0.901	–
Current employment status*						–
*Working*	6 (7.9%)	1 (2.2%)	7 (18.9%)	14 (8.9%)	**0.028**	.
*Retired/Unemployed/Student*	70 (92.1%)	44 (97.8%)	30 (81.1%)	30 (81.1%)		
Mean (SD) length of illness (years)*	15.8 (8.8)	22.0 (9.3)	20.3 (9.9)	18.5 (9.5)	**0.002**	SRP1 vs SRP2
**Mean (SD) length of stay in RF (months)**	18.8 (26.2)	42.7 (50.2)	73.4 (74.6)	39.1 (53.8)	**< 0.001**	SRP1/SRP2 vs SRP3
**Symptoms** **Mean (SD) BPRS score (range 1 - 7) Higher scores denote more severe symptoms**						
*Depression/anxiety*	2.5 (1.0)	2.1 (0.8)	2.1 (0.7)	2.3 (0.9)	**0.010**	SRP1 vs SRP2/SRP3
*Excitement*	1.8 (0.9)	1.8 (0.8)	1.7 (1.0)	1.8 (0.9)	0.690	–
*Positive symptoms*	2.4 (1.1)	2.3 (0.9)	2.5 (1.1)	2.4 (1.0)	0.849	–
*Negative symptoms*	2.3 (1.2)	2.4 (1.0)	2.0 (1.1)	2.2 (1.0)	0.296	–
*Cognitive symptoms*	1.9 (1.0)	1.6 (0.6)	1.5 (0.7)	1.8 (0.9)	**0.024**	SRP1 vs SRP3
*Total score*	2.2 (0.8) (73residents)	2.0 (0.5) (41residents)	1.9 (0.6) (31residents)	2.1 (0.7) (145residents)	0.140	–
**Negative Symptoms** **Mean (SD) BNSS score (range 0 - 6) Higher scores denote more severe symptoms**						
*Anhedonia*	2.2 (1.7)	2.4 (1.6)	1.5 (1.3)	2.1 (1.6)	**0.050**	SRP3 vs SRP2
*Distress*	2.3 (2.0)	1.6 (1.8)	1.3 (1.4)	1.9 (1.8)	**0.042**	SRP3 vs SRP1
*Asociality*	2.2 (1.7)	2.8 (1.3)	1.7 (1.5)	2.2 (1.6)	**0.017**	SRP3 vs SRP2
*Avolition*	2.1 (1.8)	2.4 (1.6)	1.6 (1.3)	2.1 (1.7)	0.087	–
*Blunted affect*	1.9 (1.8)	2.2 (1.6)	1.3 (1.5)	1.9 (1.7)	0.061	–
*Alogia*	1.7 (1.7)	1.9 (1.8)	1.0 (1.5)	1.6 (1.7)	0.054	–
*Total score*	2.0 (1.5) (72residents)	2.3 (1.2) (36residents)	1.4 (1.1) (32residents)	2.0 (1.4) (140residents)	**0.016**	SRP3 vs SRP2
**Functioning** **Mean (SD) SLOF score (range 43-215) Higher scores denote greater functioning**						
*Total score*	177.5 (27.6)	172.3 (21.1)	175.1 (18.1)	175.5 (23.8)	0.537	–

*SRP* Struttura Residenziale Psichiatrica/ Psychiatric Residential Facility

*SRP1* High intensity rehabilitation, *SRP2* Medium intensity rehabilitation, SRP3 Medium-low level support

*BPRS* Brief Psychiatric Rating Scale, *BNSS* Brief Negative Symptom Scale, *SLOF* Specific Levels of Functioning Scale

*Bold values indicate statistical significance at the *p* < 0.05 level

Residents with SSDs of different types of RF showed no differences as psychiatric severity (BPRS total mean score 2.2, SD 0.8 in SRP1; 2.0, SD 0.5 in SRP2; 1.9, SD 0.6 in SRP3; F = 2.0, df = 2, *p* = 0.140). However, SRP3 residents scored lower on the BPRS than residents of other types of RF on the depressive symptoms subscale (F = 4.7, df 2, *p* = 0.010) and the cognitive symptom subscale (F = 3.8, df 2, *p* = 0.024). They also scored lower for negative symptoms than SRP2 residents (BNSS total mean score, F = 4.2, df 2, *p* = 0.017).

There was also no statistically significant difference in psychosocial functioning as assessed by the SLOF between residents of different types of RF (total mean score SRP1 = 177.5, SD 27.6; SRP2 = 172.2, SD 21.1; SRP3 = 175.1, SD 18.1; F = 0.55, df 2, *p* = 0.537).

As shown in Table 4, residents of all three types of RF rated their functioning/disability similarly (WHODAS total mean score 1.1, SD 0.7 in SRP1; 1.0, SD 0.7 in SRP2; 0.9, SD 0.7 in SRP3; F = 0.35, df 2, *p* = 0.705).

**Table 4:** Self-rated functioning/disability, quality of life (QoL), experiences of care (therapeutic milieu and satisfaction with care) of residents of different types of RF

	SRP1 ***N =*** 76 (47.2%) residents	SRP2 ***N =*** 48 (29.8%) residents	SRP3 ***N =*** 37 (23.0%) residents	Total ***N =*** 161 (100%) residents	p ANOVA or Chi-Square* tests	Bonferroni post-hoc correction
**Functioning/disability** **Mean (SD) WHODAS 2.0 score (range 0 - 4) Higher scores denote higher disability**						
*Total score*	1.1 (0.7) (75residents)	1.0 (0.7) (47residents)	0.9 (0.7) (37residents)	1.0 (0.7) (159residents)	0.705	–
**Quality of life** **Mean (SD) WHOQOL-Brief score (range 26 -130) Higher scores denote greater QoL**						
*Physical domain*	62.9 (17.4)	69.6 (17.9)	74.1 (16.1)	67.5 (17.8)	**0.004**	SRP1 vs SRP3
*Psychological domain*	54.7 (17.7)	55.7 (18.9)	63.4 (16.9)	57.0 (18.1)	**0.048**	SRP1 vs SRP3
*Social relationship domain*	52.1 (20.3)	50.5 (20.0)	59.7 (19.8)	53.4 (20.2)	0.090	–
*Environment domain*	62.7 (16.8)	61.2 (15.8)	66.4 (15.9)	63.1 (16.3)	0.339	–
*Total score*	54.0 (17.0)	55.4 (15.9)	63.4 (17.4)	56.6 (17.1)	**0.020**	SRP1 vs SRP3
**Therapeutic milieu** **Mean (SD) WAS-P score (range 0 -100). Higher scores denote greater satisfaction**						
*Relationship dimensions*	18.1 (5.3)	16.3 (5.7)	17.4 (4.6)	17.4 (5.3)	0.212	–
*Treatment program dimensions*	21.5 (4.8)	19.8 (5.3)	20.8 (3.9)	20.9 (4.8)	0.174	–
*System maintenance dimensions*	19.8 (3.2)	19.9 (4.7)	20.6 (2.4)	20.0 (3.5)	0.518	–
*Total score*	58.6 (12.1)	56.2 (13.0)	58.8 (8.3)	58.0 (11.6)	0.500	–
**Satisfaction with the care received Mean (SD) VSSS-32 score (range 1 -5). Higher scores denote greater satisfaction**						
*Overall satisfaction*	4.1 (1.1)	3.9 (0.8)	3.8 (1.0)	4.0 (1.0)	0.239	–
*Professionals’ skills, and behaviour*	4.0 (0.8)	3.7 (0.6)	3.8 (0.7)	3.9 (0.7)	0.117	–
*Information*	3.6 (1.2)	3.4 (1.2)	3.5 (1.1)	3.5 (1.2)	0.724	–
*Access*	4.0 (0.8)	3.9 (0.9)	3.8 (0.8)	3.9 (0.8)	0.231	–
*Efficacy*	3.9 (0.8)	3.5 (0.8)	3.6 (0.8)	3.7 (0.8)	**0.031**	SRP2 vs SRP1
*Types of intervention*	3.6 (0.5)	3.5 (0.4)	3.7 (0.5)	3.6 (0.4)	0.618	–
General impression of RF	1.0 (1.8)	1.3 (1.9)	3.0 (1.9)	1.5 (2.0)	**< 0.001**	SRP1/SRP2 vs SRP3
Help received in finding a job	0.9 (1.6)	0.2 (0.7)	0.5 (1.3)	0.6 (1.4)	**0.018**	SRP2 vs SRP1
*Relatives’ involvement*	3.5 (1.0)	3.2 (1.4)	3.5 (0.9)	3.4 (1.1)	0.133	–
*Total Score*	3.8 (0.6) (65residents)	3.6 (0.5) (39residents)	3.7 (0.5) (30residents)	3.7 (0.5) (134residents)	0.281	–

*SRP* Struttura Residenziale Psichiatrica/ Psychiatric Residential Facility

*SRP1* High intensity rehabilitation, *SRP2* Medium intensity rehabilitation, *SRP3* Medium-low level support

*WHODAS 2.0* WHO Disability Assessment Schedule, *WHOQoL-Brief* WHO Quality of Life-Brief, *WAS-P* Ward Atmosphere Scale-patient version, *VSSS-32* Verona Service Satisfaction Scale 32 item

*Bold values indicate statistical significance at the *p* < 0.05 level

Mean self-rated QoL scores were highest for residents of SRP3 services (WHOQOL-Brief total mean score 54.0, SD 17.0 in SRP1; 55.4, SD 15.9 in SRP2; 63.4, SD 17.4 in SRP3 F = 2.5, df 2, *p* = 0.020).

Between RF types, there was no statistically significant difference in residents’ ratings of the therapeutic milieu where they lived (WAS-P total mean score 58.6, SD 12.1 in SRP1; 56.2, SD 13.0 in SRP2; 58.8, SD 8.3 in SRP3; F = 0.61, df =, *p* = 0.500).

Residents’ satisfaction with the care received was also similar across RF types (VSSS-32 total mean score 3.8, SD 0.6 in SRP1; 3.6, SD 0.5 in SRP2; 3.7, SD 0.5 in SRP3; F = 1.3, df = 2, *p* = 0.281), with the exception of significantly higher dissatisfaction in SRP2 residents compared to SRP1 residents for the subscales service efficacy (VSSS32, F = 3.6, df 2, *p* = 0.025), and help received in finding a job (VSSS32, F = 2.6, df 2, *p* = 0.018). The rating for ‘general impression’ was also highest amongst residents of SRP3 services (VSSS32, F = 14.2, df 2, *p* < 0.001) (see Table 4).

## Discussion

This is the first Italian study that investigated the quality of care provided in RFs and residents’ QoL and experiences of care.

We found that the RFs assessed in this study scored above 50% for all domains on an international, standardised quality assessment tool. Along with the generally positive ratings of residents about their care, our results could suggest that the quality of services provided in RFs is adequate. The QuIRC-SA domain scores of services in this study were similar (±2%) to those of a previous survey of supported accommodation in Verona [32], except for the Social Interface domain (< 4% than the Verona sample). However, it should be noted that data were collected during the Sars-cov-2 pandemic characterized by the strengthening of family relationships and increasing opportunities for community activities [53] and this fact may account for the lower scores in the Social Interface domain. However, the QuIRC-SA domain scores of the RFs in this study were lower, except for the Treatments and Interventions domain (> 2%), than those of a national sample of mental health supported accommodation services in England (mean = 69.2%, range 55.1% [SD = 8.4] to 86.7% [SD = 5.0]), the only other sample on which QuIRC-SA data have been published to date [39].

Therefore, our findings suggested room for improvement in the quality of care provided in Italian RFs, especially regarding the Recovery Based Practice. Of note, one-quarter of the RFs in our study did not provide single bedrooms, a fairly basic marker of good quality care. We also found that medium intensity rehabilitation services (SRP2) were rated lowest for quality by managers and lowest on experiences of care by residents, suggesting that these services may need particular attention.

### The weaknesses of the residential system

#### Institutional practices have not been completely erased

Despite being early adopters of deinstitutionalisation, the model of care being used by most Italian RFs has not changed for many years [36]. In the facilities where support is more intensive, the approach has been criticised for being too ‘institutionalized’ [54].

We found the average length of stay of residents of the RFs to be longer than the expected maximum length of stay described in the Ministry of Health RF typology (see table 1). Remaining in a setting that provides more support than needed can put people at risk of developing a dependency on the service rather than developing the skills and confidence to manage with less support, which can, in turn, impede the person’s potential to move on to more independent accommodation [36, 55]. This finding was previously reported in the large national survey of community facilities in Italy (PROGRES), approximately 20 years ago [16] and has also been corroborated in one of the few prospective cohort studies of RF service users in Italy [56].

We found that about 20% of RF places were unfilled, with SRP3 services having the lowest occupancy and the longest duration of stay. While this finding may indicate a lack of demand for these services, it should also be kept in mind that Italy has the lowest overall dotation of psychiatric beds as compared to all other countries of the European Union, as shown by official EUROSTAT data (see https://ec.europa.eu/eurostat/web/products-eurostat-news/-/edn-20191009-1).

### Other indicators of a lack of recovery based practice

Successful discharge from inpatient services and graduation from higher to lower supported accommodation in the community has been found to be more likely in services that provide greater recovery based practice and promotion of human rights [7, 8]. In terms of markers of recovery based practice, managers expected that only a minority of their residents were likely to move on to more independent settings. This may suggest some degree of therapeutic pessimism, contrary to one of the key components of a recovery-based approach i.e. to hold and promote hope [57]. However, this finding could simply reflect difficulties in enabling people to move on due to a lack of suitable local accommodation for them to move to, rather than a lack of belief in the possibility. Nevertheless, our finding that services were operating at around 80% occupancy suggests that a lack of availability of ‘move-on’ accommodation was unlikely to explain this, at least for moves from SPR1 to SPR2 and from SPR2 to SPR3 services.

The employment of peer support workers is a marker of a recovery-oriented service [58, 59]. Unfortunately, very few RFs in our study employed ex-service users on their staff. This kind of innovation is made difficult in Italy due to laws and regulations that restrict access to paid jobs for people with disability through prescriptive requirements that may be challenging for people with more severe mental health problems to achieve.

### Resident functioning and employment

Having a job is an important life goal for most people [32] including those with severe mental health problems [60, 61]. The low employment rate of residents we found (8.9%) was even lower than the European average for people with mental health problems (15%) [62]. The low provision of vocational therapists or IPS trainers [63, 64] and low ratings of satisfaction with the help received in searching for a job highlight that this is an area that requires improvement. It is of note that people living in SRP3 services were more likely to be employed than those living in other types of RF in our study. This group also had less severe negative symptoms which might, in part, explain this finding. It was surprising therefore that there were no statistically significant differences between service types in the staff and service user ratings of individuals’ functioning. One would expect functioning to be lowest in services offering higher support and vice versa. This might be explained by a sampling bias (i.e. service users with a similar level of functioning agreed to participate and those with lower functioning did not). Alternatively, it could be that the allocation of users to different levels of supported accommodation is not matched to the level of support available as well as it should be in the services we studied. A further possible explanation is that the measures we used did not identify more nuanced differences in functioning between residents’ groups, though this seems unlikely given that these were standardised measures.

### Limited opportunities for family support

The involvement of family members was reported by only half of the residents in our study, and we can only speculate about the reasons for this. It may be that professionals do not make adequate efforts to involve relatives in the care planning of their relative, or it may reflect estrangement secondary to the negative impact of the illness on family relationships. Whatever the reasons, the result is likely to be greater social isolation for the service user [65–67].

#### The strengths of the current system

Results discussed in the previous section suggest that although many people with severe mental health problems are living in the community in Italy, the RFs in which they live may be less enabling of people’s rehabilitation and recovery than one would expect [24, 32, 36, 54]. Nevertheless, we also identified considerable strengths. Indeed, we found that the service quality domain that scored highest across the RFs in this study was the promotion of human rights, an aspect of care previously noted to be associated with better outcomes [7].

Empowerment and self-determination, which include having adequate knowledge of own rights as a human being and as a person with a disability [68], and the right to determine own destiny [69] are key components of personal recovery. The high ratings we found for services concerning the promotion of Human Rights suggest that Italian RFs are doing well in this regard.

We also found that almost all RFs used individualised care plans suggesting person-centred programmes of care were being delivered. Moreover, the positive ratings of quality by managers and residents regarding the built environment, the therapeutic culture and the care received, particularly in the most supported residential facilities (SRP1) are in line with previous studies which concluded that RFs with a high intensity of care provide homely and safe environments for people with severe mental health problems [36, 55]. SRP1 is the first step in the care pathway enabling users to acquire essential skills to take responsibility for their lives and improve their overall situation [32].

Given that residents of SRP3 had the lowest severity of psychopathology, it is perhaps no surprise that these services scored best for quality. What we do not know from this cross-sectional study is whether the quality of care drives resident outcomes such as quality of life and functioning, or whether services that work with those with more severe problems (who are therefore more likely to be living in higher supported settings) have greater difficulty in delivering better quality care as a result.

#### Strengths and limitations

Our sample includes RFs which voluntarily agreed to take part in this project. One significant limitation in the interpretation of our findings is the sampling bias. Our sample of 161 residents represents at most around one-third of all those living in the RFs recruited for this study. Although we focused on residents with a diagnosis of SSD who make up the majority of those living in RFs [20, 22] we had excluded those who for various reasons were unable to participate in the research study. Thus, our results may not represent people with other diagnoses or more severe symptoms or cognitive impairments. This was a cross-sectional survey and our models can therefore only identify associations between variables, without infer causality. Finally, data were collected during the sars-cov-2 pandemic, which influenced routine activities and clinical practices [70].

## Conclusions

This study aimed to investigate the quality of care provided by RFs in Italy and residents’ experiences of care. Our findings suggest that the overall quality of services is lower than similar services in England [30]. Further efforts should be made to improve adherence to international guidelines with a particular focus on ensuring that staff would adopt a recovery orientation that might enhance service users’ experiences of care and facilitate progress in their rehabilitation.

## Supplementary Information

**Additional file 1.**

## Data Availability

The datasets used and/or analysed during the current study are available on ZENODO with a 2 year embargo (from today until 14 April 2024). 10.5281/zenodo.6497621

## Abbreviations

BNSS: Brief Negative Symptom Scale
BPRS: Brief Psychiatric Rating Scale
QuIRC-SA: Quality Indicator for Rehabilitative Care - Supported Accommodation
RFs: Residential facilities
SLOF: Specific Levels of Functioning Scale
SRP: Struttura Residenziale Psichiatrica/ Psychiatric Residential Facility
VSSS-32: Verona Service Satisfaction Scale
WAS-P: Ward Atmosphere Scale-patient version
WHODAS 2.0: World Health Organization Disability Assessment Schedule
WHOQoL-Bref: World Health Organization Quality of Life-Bref
