# Supplementary material for: Quality of residential facilities in Italy: satisfaction and quality of life of residents with schizophrenia spectrum disorders
Source: BMC Psychiatry. 2022 Nov 18;22:717. doi: 10.1186/s12888-022-04344-w (PMC9672559; doi:10.1186/s12888-022-04344-w)
Supplement: Supplementary file 1 — Additional file 1. [file 12888_2022_4344_MOESM1_ESM.docx]

**SUPPLEMENTARY TABLE 1**

**Characteristics of 48 Italian RFs, by RF type (classification of the Italian Ministry of Health).**

|  | **SRP1**  **17 (35.4%)**  **RFs** | **SRP2**  **15 (31.3%)**  **RFs** | **SRP3**  **16 (33.3%)**  **RFs** | **Total RFs**  **48 (100%)** | ***p***  ***ANOVA or Chi-Square* tests*** | **Bonferroni post-hoc** |
| --- | --- | --- | --- | --- | --- | --- |
| **RF characteristics** |  |  |  |  |  |  |
| Number of years RF open in current form, Mean (SD) [Range] | 19.5 (8.8) [1-40]  (15) | 15.3 (8.0) [2-28] | 11.9 (7.4) [1-25] | 15.5 (8.5) [1-40]  (46) | **0.039** | SRP3 vs SRP1 |
| Service location |  |  |  |  |  |  |
| *In the inner city* | 5 (29.4%) | 8 (57.1%) | 9 (56.3%) | 22 (46.8%) | 0.284 | - |
| *In the suburbs* | 11 (64.7%) | 4 (28.6%) | 6 (37.5%) | 21 (44.7%) |  |  |
| *In the country* | 1 (5.9%) | 2 (14.3%) | 1 (6.3%) | 4 (8.5%) |  |  |
| Number of males, Mean (SD) | 8.1 (2.4) | 10.2 (3.5) | 6.1 (4.4) | 8.0 (3.9) | **0.011** | SRP3 vs SRP2 |
| Number of females, Mean (SD) | 5.9 (3.0) | 4.8 (3.6) | 2.9 (3.1) | 4.6 (3.4) | **0.034** | SRP3 vs SRP1 |
| Total beds/places, Mean (SD) [Range] | 16.9 (3.4) [12-20] | 16.1 (4.9) [6-20] | 9.3 (6.9) [3-29] | 14.1 (6.2) [3-29] | **<0.001** | SRP3 vs SRP1/SRP2 |
| Beds/places currently filled, Mean (SD), [Range] | 14.5 (3.1) [10-20] | 14.9 (4.4) [6-20] | 9.0 (7.0) [2-29] | 12.8 (5.7) [2-29] | **<0.001** | SRP3 vs SRP1/SRP2 |
| Rate of occupancy, N (%) | 14.4 (85.8) | 14.9 (92.5) | 5.8 (62.4) | 10.2 (79.8) | **<0.001** | SRP3 vs SRP1/SRP2 |
| Single bedrooms, N (%) | 16 (94%) | 9 (60%) | 9 (56.3%) | 34 (70.8%) | - | - |
| **Staffing** |  |  |  |  |  |  |
| Psychiatrist | 16 (94.1%) | 15 (100%) | 12 (75%) | 43 (89.6%) | 0.056 |  |
| Clinical psychologist | 13 (76.5%) | 14 (93.3%) | 8 (50%) | 35 (72.9%) | **0.023** | SRP3 vs SRP1/SRP2 |
| TeRP (similar to Occupational therapist) | 11 (64.7%) | 10 (66.7%) | 6 (37.5%) | 27 (56.3%) | 0.179 |  |
| Nurse | 15 (88.2%) | 13 (86.7%) | 10 (62.5%) | 38 (79.2%) | 0.132 |  |
| Support worker | 14 (82.4%) | 12 (80.0%) | 13 (81.3%) | 39 (81.3%) | 0.986 |  |
| Social worker | 9 (52.9%) | 10 (66.7%) | 5 (31.3%) | 24 (50.0%) | 0.137 |  |
| Counsellor psychotherapist | 6 (35.3%) | 4 (26.7%) | 4 (25%) | 14 (29.2%) | 0.783 |  |
| Vocational therapist (e.g. the Individual Placement and Support - IPS trainer) | 5 (29.4%) | 2 (14.3%) | 2 (12.5%) | 9 (19.1%) | 0.401 |  |
| Ex-service user employed as a member of staff | 1 (5.9%) | 2 (13.3%) | 2 (12.5%) | 5 (10.4%) | 0.746 |  |
| **Rehabilitation programme** |  |  |  |  |  |  |
| Individualised care plans | 17 (100.0%) | 13 (92.9%) | 16 (100%) | 46 (97.9%) |  |  |
| All patients have allocated key-worker | 17 (100.0%) | 14 (93.3%) | 16 (100%) | 47 (97.8%) | 0.300 |  |
| Number of families currently involved in users care, N (%) | 8.5 (50.3) | 7.3 (45.3) | 4.8 (51.6) | 6.9 (48.9) | 0.140 |  |
| Expected maximum length of stay (years) by law, Mean (SD) [Range] | 2.3 (0.8) [1-4] | 2.8 (1.0) [1-5] | 2.9 (1.0) [2-5] | 2.7 (1.0) [1-5] | 0.169 | - |
| Average length of stay (years), Mean (SD) [Range] | 2.4 (0.5) [2-3] | 3.5 (1.2) [3-6] | 2.5 (0.6) [2-3] | 2.9 (1.0) [2-6] | **0.020** | SRP1 vs SRP2 |
| Move on to more independent living |  |  |  |  |  |  |
| *Hopeful users will move on* | 3.9 (26.9) | 4.0 (26.8) | 4.2 (46.7) | 4.0 (31.3) | 0.549 |  |
| *Number of users that will move on* | 3.2 (22.1) | 2.3 (15.4) | 2.8 (31.1) | 2.8 (21.9) | 0.060 |  |
| *Bold values indicate statistical significance at the p < 0.05 level  SRP = Struttura Residenziale Psichiatrica/ Psychiatric Residential Facility  SRP1 = High intensity rehabilitation; SRP2 = Medium intensity rehabilitation; SRP3 = Medium-low level support | | | | | | |
